# Greater HbA_1c_ variability is associated with increased cardiovascular events in type 2 diabetes patients with preserved renal function, but not in moderate to advanced chronic kidney disease

**DOI:** 10.1371/journal.pone.0178319

**Published:** 2017-06-07

**Authors:** Mei-Yueh Lee, Pi-Jung Hsiao, Yu-Ting Huang, Jiun-Chi Huang, Wei-Hao Hsu, Szu-Chia Chen, Shyi–Jang Shin

**Affiliations:** 1 Graduate Institute of Clinical Medicine, College of Medicine, Kaohsiung Medical University, Kaohsiung, Taiwan; 2 Division of Endocrinology and Metabolism, Department of Internal Medicine, Kaohsiung Medical University Hospital, Kaohsiung Medical University, Kaohsiung, Taiwan; 3 Department of Internal Medicine, Kaohsiung Municipal Hsiao-Kang Hospital, Kaohsiung Medical University, Kaohsiung, Taiwan; 4 Faculty of Medicine, College of Medicine, Kaohsiung Medical University, Kaohsiung, Taiwan; 5 Division of Medical Statistics and Bioinformatics, Department of Medical Research, Kaohsiung Medical University Hospital, Kaohsiung Medical University, Kaohsiung, Taiwan; 6 Division of Nephrology, Department of Internal Medicine, Kaohsiung Medical University Hospital, Kaohsiung Medical University, Kaohsiung, Taiwan; 7 Center for Lipid and Glycomedicine Research, Kaohsiung Medical University, Kaohsiung, Taiwan; Weill Cornell Medical College in Qatar, QATAR

## Abstract

Emerging evidence suggests that glycemic variability may be a more reliable measure of glycemic control than mean HbA_1c_ in type 2 diabetes mellitus. This study aimed to determine if HbA_1c_ variability is associated with cardiovascular events in type 2 diabetic patients and if different renal functions affect such association. This longitudinal study enrolled 8259 diabetic patients from the Kaohsiung Medical University Research Database in 2009 and were followed-up until 2015. Intra-individual HbA_1C_ variability was defined as the standard deviation (SD) of HbA_1c_ and cardiovascular events were defined as hospitalization for coronary artery disease, unstable angina, myocardial infarction, stroke, peripheral artery disease, and cardiovascular death. The patients were grouped into two based on their estimated glomerular filtration rate (eGFR) ≥ 60 or < 60 min/ml/1.73m^2^. In a mean follow-up period of 6.3 years, cardiovascular events were recorded in 8.9% of the patients. In an adjusted Cox model, high HbA_1c_ SD (hazard ratio, 1.290; 95% confidence interval, 1.008–1.650; *p* = 0.043), but not mean HbA_1c_, was associated with significantly increased risk of cardiovascular events in patients with eGFR ≥ 60 min/ml/1.73m^2^. This association was not seen in patients with eGFR < 60 min/ml/1.73m^2^. In this study, greater HbA_1c_ variability is associated with increased risk of cardiovascular among patients with preserved renal function, but not in those with moderate to advanced chronic kidney disease.

## Introduction

There is a growing number of people with diabetes mellitus (DM) worldwide. People with diabetes have a two- to three-fold increased risk of developing cardiovascular disease, which remains the major cause of death in such patients [1, 2]. Several studies have demonstrated the importance of time-averaged mean levels of glycemia, as measured by glycosylated hemoglobin (HbA_1c_), which has since been considered the “gold standard” for measuring glycemic control [3, 4]. However, recent research has pointed that plasma glucose variability, regardless of hyperglycemia level, may confer additional risk for the development of micro- and macrovascular diabetic complications [5–7].

Among studies on type 2 DM with preserved renal function, glucose variability is positively associated with the development of diabetic retinopathy, neuropathy, and nephropathy [8–11], but its effects on macrovascular events remain uncertain. Wan et al. evaluated the association of HbA_1c_ variability with cardiovascular events in patients with type 2 DM and found that high variability was associated with increased cardiovascular events [12]. On the other hand, the cross-sectional analysis within the Italian Renal Insufficiency and Cardiovascular Events (RIACE) multi-center study showed no impact of HbA_1c_ variability [11]. Thus, current literature is conflicting and limited, especially on different renal functions.

Even less is known on how glycemic control affects prognosis in diabetic patients with late stage chronic kidney disease (CKD), especially since these patients are often excluded from clinical trials. Few studies have evaluated the association of HbA_1c_ variability with cardiovascular events in diabetic patients with moderate-to-advanced CKD. This study therefore aimed to assess whether HbA_1c_ variability is associated with cardiovascular events in type 2 DM patients. The study also tested whether different renal functions affect such an association.

## Subjects and methods

### Setting

This study used data from the Kaohsiung Medical University Hospital Research Database (KMUHRD). Kaohsiung Medical University Hospital (KMUH), established in 1957, is a medical center with 1600 beds, 1200 general beds, 144 intensive care beds, and about 6000 clinical visits per day in 2015. The KMUHRD includes data for approximately 2,000,000 patients who attended KMUH in the period 2009–2015, with predominance from southern Taiwan.

The KMUHRD offers a comprehensive database with coverage on ambulatory care, hospital admissions, dental services, drug-dispensing records, and biochemical data. The hospital records include detailed information on primary and secondary diagnoses, procedures, and dates of hospital admission and discharge. All diagnoses are coded according to the International Classification of Diseases, 9th Revision, Clinical Modification (ICD-9-CM). The drug-dispensing histories contain data on the dispensed drug, prescriber type, dispensing date, dispensed amount, prescribed dose regimens, and duration of use (prescription length).

The database is managed by the KMUH Division of Medical Statistics and Bioinformatics. For confidentiality and in accordance with the Personal Information Protection Act, all personal identifiers were removed and the authorized researchers only performed data linkage, processing, and statistical analyses with specified computers in an independent 24-hr monitored room using encrypted identifiers. All data analysts were required to sign an agreement. Only tables and figures from statistical analysis were allowed to be released after inspection.

### Study population

**Fig 1:**
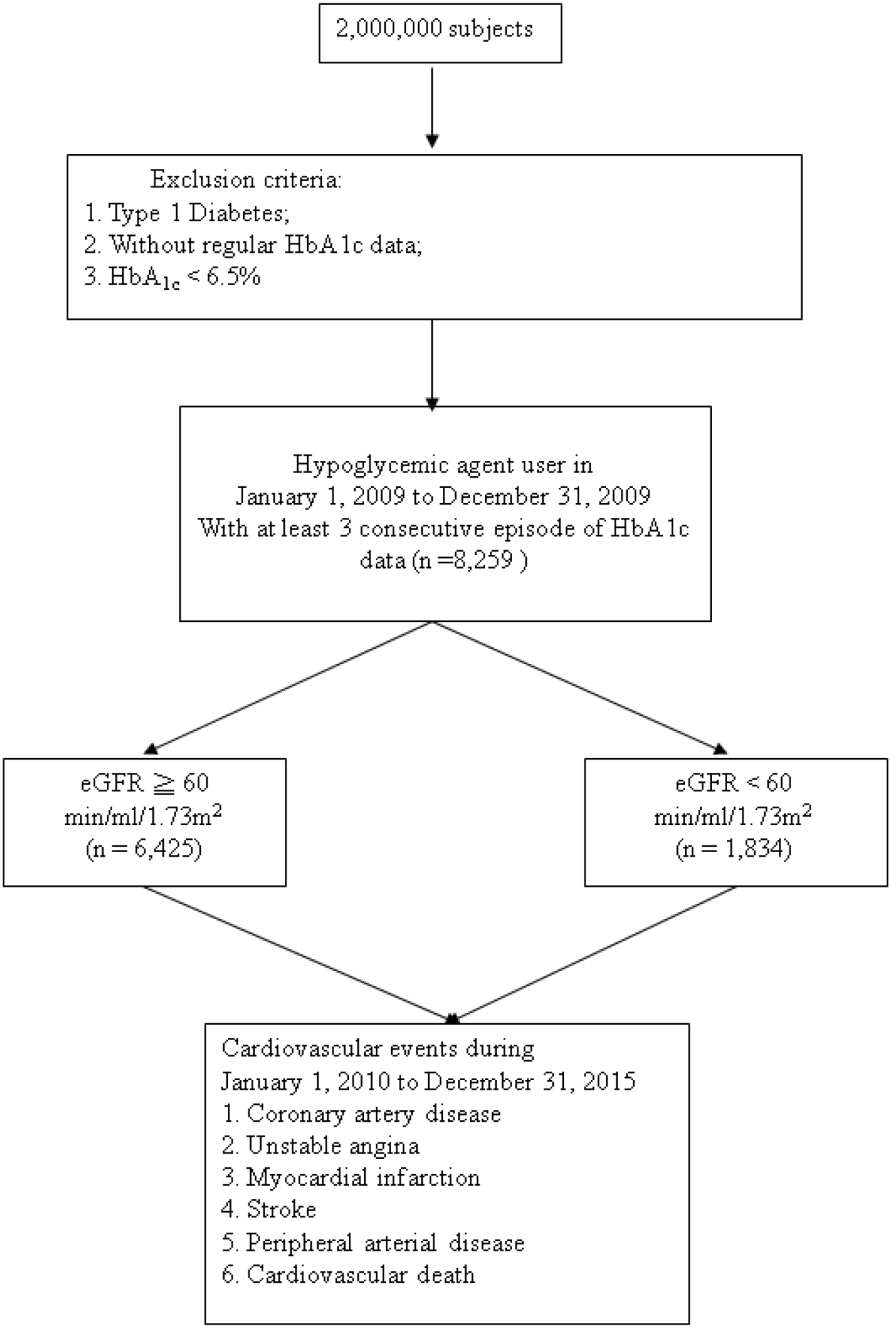
Flow chart of study participants for the study to evaluate the effect of SD of HbA_1c_ on cardiovascular events in type 2 diabetes.

The entry date was between January 1, 2009 and December 31, 2009. All patients with type 2 diabetes (ICD-9-CM code 250.1–250.9) on hypoglycemic agents and with an HbA_1c_ level of ≥ 6.5% were enrolled and followed-up from January 1, 2010 until December 31, 2015. Data on the occurrence of cardiovascular events, including coronary artery disease (ICD-9-CM code 414), myocardial infarction (ICD-9-CM code 410–412), ischemic stroke (ICD-9-CM code 435–438), and peripheral arterial disease (ICD-9-CM code 443 and 25070) on follow-up were also obtained from the KMUHRD. The exclusion criteria were type 1 diabetes, mortality, DM diagnosis of after a cardiovascular event, use of insulin dependent and independent agents, and HbA_1c_ < 6.5%. The study patients were then stratified into two groups based on their estimated glomerular filtration rate (eGFR) ≥ 60 and < 60 min/ml/1.73m^2^ (Fig 1). We classified our patients with evidence of kidney damage lasting for more than 3 months into CKD stage 3, 4, and 5, based on estimated eGFR level (mL/min/1.73 m^2^) of 30 to 59, 15 to 29, and < 15, respectively.

The data recorded before entry into the study were age, sex, duration of diabetes, hypertension (ICD-9-CM code 401 and 405), hyperlipidemia (ICD-9-CM code 272), nephropathy (ICD-9-CM code 580–589), retinopathy (ICD-9-CM code 250.50), and neuropathy (ICD-9-CM code 249.60 and 250.60). Urine albumin and creatinine were measured on a spot urine sample by an autoanalyzer (COBAS Integra 400 plus; Roche Diagnostics, North America) and nephropathy was defined as the ratio of urine albumin to creatinine of ≥ 30 mg/g.

### Definition of study endpoint

The study endpoint was defined as cardiovascular events, including coronary artery disease, stroke, peripheral arterial disease, and cardiovascular death. Coronary artery disease was defined as a history of angina and ischemic electrocardiogram change, ST-elevation myocardial infarction, non ST-elevation myocardial infarction, unstable angina or having undergone coronary bypass surgery or angioplasty.

### Ethics statement

The study protocol was approved by the Institutional Review Board of Kaohsiung Medical University Hospital (KMUHIRB-E(I)-20160032). Written informed consent was obtained from the patients, and all clinical investigations were conducted according to the principles expressed in the Declaration of Helsinki. The patients also consented to the publication of the clinical details.

### HbA_1c_ variability

The intra-personal mean and standard deviation (SD) of HbA_1c_ was calculated for each patient, and the latter was considered an index of HbA_1c_ variability. The estimated HbA_1c_ variability was based on at least three HbA_1c_ measurements.

### Statistical analysis

All statistical analyses were performed using the SPSS 19.0 for Windows (SPSS Inc. Chicago, USA). Data were expressed as percentages or mean ± standard deviation. Between-group differences were assessed using the chi-square test for categorical variables and the independent t-test for continuous variables. The association between HbA_1c_ variability and cardiovascular events was assessed using multivariate Cox proportional hazard analysis. Age, sex, and significant variables in the univariate analysis were used in the multivariate analysis. The Kaplan-Meier analysis was used to determine the survival curve for cardiovascular events. Statistical significance was set at *p*<0.05.

## Results

Comparisons of baseline data between patients with eGFR ≥ and those with < 60 min/ml/1.73m^2^ were show (Table 1). The patients’ mean age was 62.0 ± 11.9 years. Compared to patients with eGFR ≥ 60 min/ml/1.73m^2^, patients with eGFR < 60 min/ml/1.73m^2^ were older and had higher prevalence rates of hypertension, dyslipidemia, diabetic retinopathy, neuropathy, and nephropathy. They had higher HbA_1c_ SD, triglyceride, and total cholesterol levels, but lower mean HbA_1c_, high-density lipoprotein (HDL) cholesterol, and eGFR. They also had higher percentage use of angiotensin converting enzyme inhibitors (ACEIs) and/or angiotensin II receptor blockers (ARBs), aspirin, and insulin. In addition, patients with eGFR < 60 min/ml/1.73m^2^ had less follow-up days and a higher frequency of cardiovascular events (*p* < 0.001).

**Table 1:** Comparison of clinical characteristics according to eGFR ≧ or < 60 min/ml/1.73m^2^.

Characteristics	eGFR ≧ 60 min/ml/1.73m^2^ (n = 6425)	eGFR < 60 min/ml/1.73m^2^ (n = 1834)	All (n = 8259)	*p*
Age (year)	60.3 ± 11.4	68.6 ± 10.9	62.0 ± 11.9	< 0.001
Male gender (%)	51.5	54.0	52.0	0.056
Hypertension (%)	69.4	84.9	72.8	< 0.001
Dyslipidemia (%)	71.4	61.8	69.3	< 0.001
Retinopathy (%)	3.6	11.4	5.3	< 0.001
Neuropathy (%)	12.2	17.4	13.4	< 0.001
Nephropathy (%)	3.9	10.7	5.4	< 0.001
DM duration > 5 years (%)	91.7	92.6	91.9	0.215
Laboratory parameters				
SD HbA_1C_ (%)	0.81 ± 0.56	0.93 ± 0.65	0.84 ± 0.58	< 0.001
Mean HbA_1C_ (%)	7.5 ± 1.2	7.4 ± 1.2	7.5 ± 1.2	0.017
Triglyceride (mg/dL)	146.7 ± 136.8	161.6 ± 114.3	154.7 ± 206.0	< 0.001
Total cholesterol (mg/dL)	w176.8 ± 41.0	181.0 ± 48.1	178.2 ± 44.9	0.001
HDL-cholesterol (mg/dL)	41.3 ± 12.4	38.6 ± 12.4	40.7 ± 12.5	< 0.001
LDL-cholesterol (mg/dL)	102.8 ± 33.1	102.2 ± 36.3	102.6 ± 34.0	0.578
eGFR (mL/min/1.73 m^2^)	96.0 ± 23.5	39.9 ± 14.8	84.5 ± 35.4	< 0.001
Medications				
ACEI and/or ARB use (%)	62.8	80.5	66.6	< 0.001
Aspirin use (%)	29.1	42.7	32.1	< 0.001
Statin and/or fibrate use (%)	69.6	70.8	69.9	0.341
Insulin use (%)	18.1	35.3	22.1	< 0.001
Days of follow-up (days)	2330.4 ± 426.7	2165.6 ± 650.1	2294.3 ± 489.1	< 0.001
CV events (%)	6.5	17.6	8.9	< 0.001

Abbreviations. eGFR, estimated glomerular filtration rate; DM, diabetes mellitus; SD, standard deviation; HDL, high-density lipoprotein; LDL, low-density lipoprotein; ACEI, angiotensin converting enzyme inhibitor; ARB, angiotensin II receptor blocker; CV, cardiovascular.

In a mean follow-up period of 6.3 years, cardiovascular events were recorded in 8.9% of the patients. These included hospitalization for coronary artery disease (n = 876), stroke (n = 100), peripheral artery disease (n = 54), and cardiovascular death (n = 27).

### Risk of cardiovascular events in patients with eGFR ≥ 60 min/ml/1.73m^2^

Cox proportional hazard regression analysis was used to evaluate the association between SD of HbA_1c_ and cardiovascular events in patients with eGFR ≥ 60 min/ml/1.73m^2^ (Table 2). By univariate regression analysis, old age, male sex, hypertension, diabetic retinopathy and neuropathy, high HbA_1c_ SD, high mean HbA_1c_, high triglyceride, low HDL-cholesterol, low eGFR, ACEI and/or ARB use, aspirin use, statin and/or fibrate use, and insulin use were associated with significantly higher risk of cardiovascular events.

**Table 2:** Risk factors for cardiovascular events using Cox proportional hazards model in patients with eGFR ≧ 60 min/ml/1.73m^2^.

	Univariable		Multivariable	
Parameters	HR (95% CI)	*p*	HR (95% CI)	*p*
Age (per 1 year)	1.044 (1.035–1.053)	< 0.001	1.043 (1.030–1.057)	< 0.001
Male gender	1.288 (1.063–1.561)	0.010	1.427 (1.112–1.832)	0.005
Hypertension	1.829 (1.441–2.322)	< 0.001	1.010 (0.744–1.372)	0.947
Dyslipidemia	1.167 (0.939–1.451)	0.164	–	–
Retinopathy	1.739 (1.170–2.586)	0.006	1.136 (0.636–2.028)	0.666
Neuropathy	1.462 (1.133–1.887)	0.003	0.988 (0.703–1.388)	0.945
Nephropathy	0.902 (0.539–1.510)	0.694	–	–
DM duration > 5 years	1.167 (0.805–1.691)	0.415	–	–
Laboratory parameters				
SD HbA_1C_ (per 1%)	1.427 (1.242–1.640)	< 0.001	1.290 (1.008–1.650)	0.043
Mean HbA_1C_ (per 1%)	1.115 (1.036–1.199)	0.004	0.941 (0.825–1.074)	0.365
Triglyceride (per 1 mg/dL)	1.001 (1.001–1.001)	< 0.001	1.001 (1.000–1.001)	0.010
Total cholesterol (per 1 mg/dL)	1.001 (0.998–1.003)	0.499	–	–
HDL-cholesterol (per 1 mg/dL)	0.983 (0.973–0.993)	0.001	0.992 (0.982–1.004)	0.182
LDL-cholesterol (per 1 mg/dL)	0.998 (0.995–1.001)	0.281	–	–
eGFR (per 1 mL/min/1.73 m^2^)	0.986 (0.982–0.991)	< 0.001	0.999 (0.993–1.005)	0.775
Medications				
ACEI and/or ARB use	2.483 (1.956–3.152)	< 0.001	1.818 (1.316–2.510)	< 0.001
Aspirin use	4.701 (3.856–5.732)	< 0.001	3.716 (2.893–4.773)	< 0.001
Statin and/or fibrate use	1.861 (1.462–2.367)	< 0.001	1.623 (1.172–2.247)	0.004
Insulin use	1.757 (1.421–2.174)	< 0.001	1.342 (0.995–1.811)	0.054

Values expressed as Hazard Ratios and 95% confidence interval (CI).

By multivariate analysis, old age (hazard ratio [HR], 1.043; 95% confidence interval [CI], 1.030–1.057; *p* < 0.001), male sex (HR, 1.427; 95% CI, 1.112–1.832; *p* = 0.005), high HbA_1c_ SD (HR, 1.290; 95% CI, 1.008–1.650; *p* = 0.043), high triglyceride (HR, 1.001; 95% CI, 1.000–1.001; *p* = 0.010), ACEI and/or ARB use (HR, 1.818; 95% CI, 1.316–2.510; *p* < 0.001), aspirin use (HR, 3.716; 95% CI, 2.893–4.773; *p* < 0.001), and statin and/or fibrate use (HR, 1.623; 95% CI, 1.172–2.247; *p* = 0.004) were associated with significantly increased risk of cardiovascular events.

**Fig 2:**
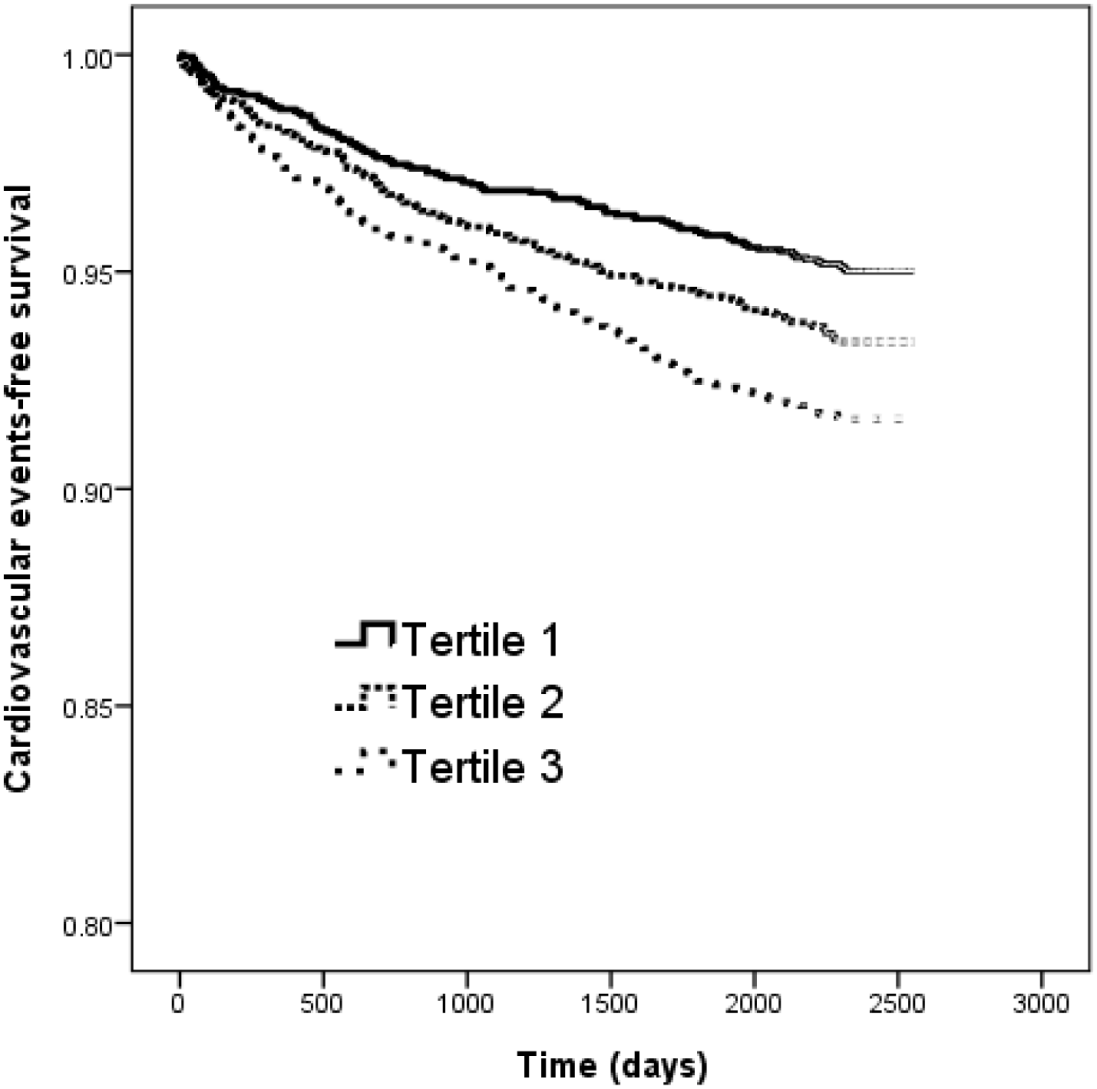
Kaplan-Meier analysis of cardiovascular events-free survival according to tertile of SD of HbA_1c_ (log-rank *p* < 0.001) in patients with eGFR ≧ 60 min/ml/1.73m^2^. Diabetic patients with tertile 2 and tertile 3 of HbA_1c_ SD had a worse cardiovascular events-free survival than those with tertile 1 of HbA_1c_ SD.

The Kaplan-Meier curves for cardiovascular event-free survival (log-rank *p* < 0.001) of study patients with eGFR ≥ 60 min/ml/1.73m^2^ according to tertiles of SD of HbA_1C_ (<0.48 and ≥ 0.48 to < 0.90 and ≥ 0.90%) revealed that tertile 2 (HR, 1.341; 95% CI, 1.047–1.705; *p* = 0.023) and tertile 3 (HR, 1.724; 95% CI, 1.355–2.191; *p* < 0.001) patients had worse cardiovascular events-free survival than those in tertile 1 (Fig 2).

We further performed the analysis of relation of SD of HbA_1C_ for separate cardiovascular events in patients with eGFR ≧ 60 min/ml/1.73m^2^, and found that high HbA_1c_ SD (HR, 1.353; 95% CI, 1.024–1.787; *p* = 0.033) was still associated with significantly increased risk of coronary artery disease after multivariate analysis (Table 3).

**Table 3:** Relation of SD of HbA_1C_ for separate cardiovascular events using Cox proportional hazards model in patients with eGFR ≧ 60 min/ml/1.73m^2^.

	Univariable		Multivariable	
Parameters	HR (95% CI)	*p*	HR (95% CI)	*p*
Coronary artery disease	1.415 (1.212–1.652)	< 0.001	1.353 (1.024–1.787)	0.033
Stroke	1.140 (0.781–1.664)	0.496	1.606 (0.894–2.887)	0.113
Peripheral artery disease	2.054 (1.326–3.182)	0.001	1.253 (0.477–3.296)	0.647

Values expressed as Hazard Ratios and 95% confidence interval (CI). Adjusted for age, gender, a history of hypertension, retinopathy and neuropathy, SD of HbA_1C_, mean HbA_1C_, triglyceride, HDL-cholesterol, eGFR, and medications use, including ACEI and/or ARB, aspirin, statin and/or fibrate, and insulin.

### Risk of cardiovascular events in patients with eGFR < 60 min/ml/1.73m^2^

The Cox proportional hazard regression was used to evaluate the association between SD of HbA_1c_ and cardiovascular events in patients with eGFR < 60 min/ml/1.73m^2^ (Table 4). By univariate regression analysis, old age, female sex, hypertension, diabetic retinopathy and nephropathy, high HbA_1c_ SD, high triglyceride, low HDL-cholesterol, low eGFR, ACEI and/or ARB use, aspirin use, statin and/or fibrate use, and insulin use were associated with significantly greater risk of cardiovascular events.

**Table 4:** Risk factors for cardiovascular events using Cox proportional hazards model in patients with eGFR < 60 min/ml/1.73m^2^.

	Univariable		Multivariable	
Parameters	HR (95% CI)	*p*	HR (95% CI)	*p*
Age (per 1 year)	1.009 (0.998–1.019)	0.102	1.007 (0.992–1.022)	0.378
Male gender	0.858 (0.687–1.070)	0.174	0.729 (0.534–0.994)	0.046
Hypertension	1.825 (1.246–2.675)	0.002	1.613 (0.889–2.925)	0.115
Dyslipidemia	1.146 (0.909–1.445)	0.249	–	–
Retinopathy	1.799 (1.344–2.406)	< 0.001	1.420 (0.915–2.203)	0.118
Neuropathy	0.999 (0.745–1.339)	0.995	–	–
Nephropathy	0.590 (1.169–2.162)	0.003	1.294 (0.818–2.045)	0.271
DM duration > 5 years	0.826 (0.553–1.235)	0.352	–	–
Laboratory parameters				
SD HbA_1C_ (per 1%)	1.211 (1.041–1.410)	0.013	1.014 (0.803–1.280)	0.909
Mean HbA_1C_ (per 1%)	1.071 (0.978–1.173)	0.140	–	–
Triglyceride (per 1 mg/dL)	1.001 (1.001–1.002)	0.001	1.000 (0.999–1.002)	0.648
Total cholesterol (per 1 mg/dL)	1.001 (0.998–1.003)	0.540	–	–
HDL-cholesterol (per 1 mg/dL)	0.981 (0.968–0.994)	0.004	0.977 (0.964–0.992)	0.002
LDL-cholesterol (per 1 mg/dL)	1.000 (0.996–1.003)	0.810	–	–
eGFR (per 1 mL/min/1.73 m^2^)	0.976 (0.969–0.983)	< 0.001	0.983 (0.972–0.993)	0.001
Medications				
ACEI and/or ARB use	1.562 (1.132–2.155)	0.007	1.584 (0.998–2.516)	0.051
Aspirin use	2.455 (1.953–3.085)	< 0.001	2.882 (2.081–3.990)	< 0.001
Statin and/or fibrate use	1.493 (1.145–1.948)	0.003	1.342 (0.890–2.023)	0.160
Insulin use	1.885 (1.510–2.352)	< 0.001	1.417 (1.020–1.969)	0.038

Values expressed as Hazard Ratios and 95% confidence interval (CI).

In the multivariate analysis, female sex (HR, 0.729; 95% CI, 0.534–0.994; *p* = 0.046), low HDL-cholesterol (HR, 0.977; 95% CI, 0.964–0.992; *p* = 0.002), low eGFR (HR, 0.983; 95% CI, 0.972–0.993; *p* = 0.001), aspirin use (HR, 2.882; 95% CI, 2.081–3.990; *p* < 0.001), and insulin use (HR, 1.417; 95% CI, 1.020–1.969; *p* = 0.038) were associated with significantly higher risk of cardiovascular events. However, HbA_1c_ SD was not.

We further performed the analysis of relation of SD of HbA_1C_ for cardiovascular events in patients with different CKD stage, and found that no significant association was noted in each CKD stage after multivariate analysis (Table 5).

**Table 5:** Relation of SD of HbA_1C_ for cardiovascular events using Cox proportional hazards model in patients with different CKD stage.

	Univariable		Multivariable	
Parameters	HR (95% CI)	*p*	HR (95% CI)	*p*
CKD stage 3	1.323 (1.085–1.614)	0.006	1.086 (0.800–1.474)	0.598
CKD stage 4	0.977 (0.711–1.363)	0.892	1.215 (0.782–1.888)	0.387
CKD stage 5	1.086 (0.804–1.466)	0.591	1.619 (0.902–2.908)	0.107

Values expressed as Hazard Ratios and 95% confidence interval (CI). Adjusted for age, gender, a history of hypertension, retinopathy and nephropathy, SD of HbA_1C_, triglyceride, HDL-cholesterol, eGFR, and medications use, including ACEI and/or ARB, aspirin, statin and/or fibrate, and insulin.

## Discussion

This study investigated the association between HbA_1c_ variability and cardiovascular events in patients with type 2 DM over a follow-up period of 6.3 years. The results show that patients with greater HbA_1c_ variability have increased risk of cardiovascular events in diabetic patients with eGFR ≥ 60 min/ml/1.73m^2^, but not in those with eGFR < 60 min/ml/1.73m^2^.

The first important finding here is that among diabetic patients with higher eGFR, greater HbA_1c_ variability is associated with increased risk for cardiovascular events, independent of mean HbA_1c_ levels. In a subsequent *post hoc* analysis, the Diabetes Control and Complications Trial (DCCT) data investigated the relationship between the risk of progression of retinopathy and HbA_1c_ levels after 5 to 9 years of follow-up [13]. Their results revealed that the patients who received intensive treatment had a significantly lower risk of the progression of retinopathy than those with conventional treatment despite comparable HbA_1c_ levels. The authors concluded that the mean level of HbA_1c_ may not be the key factor determining the degree of hyperglycemia, and that other risk factors independent of HbA_1c_ level may increase the risk of complications from diabetes due to additional metabolic burden [13]. Such findings may be partly due to the fact that studies have used HbA_1c_ as a marker of glucose control, which fails to reflect glucose variability and the risk associated with extreme glucose change over an extended time period. The association between HbA_1c_ variability and the development of cardiovascular events may be due to several mechanisms. In an *in vitro* study, Risso et al. reported that fluctuations in glycemia have a more harmful effect on endothelial cells than a persistently high level of glucose. They also found that apoptosis was significantly higher in human umbilical vein endothelial cells incubated in media in which the glucose concentration was alternately set at 5 or 20 mmol/l every other day for 14 days compared to a fixed high concentration of glucose [14]. Furthermore, intermittently high glucose levels enhance the formation of markers of oxidative stress, leading to increased cellular apoptosis [15]. In an *in vivo* study, Horva´th et al. reported that rats intermittently treated with glucose had a significantly worse endothelial function than those that were untreated despite a lower total exposure to glucose. Furthermore, the authors also hypothesized that the poly(ADP-ribose) polymerase pathway had been activated [16]. Human studies also showed that acute glucose fluctuations strongly correlated with triggering oxidative stress, urinary levels of 8-iso PGF2*α*, and more endothelial function damage leading to higher levels of oxidative stress markers than constantly high glucose [17, 18]. Some studies reveal that glucose variability is associated with the risk of hypoglycemia [19, 20], which, in turn, may contribute to increased progression of cardiovascular disease and mortality by inducing inflammation, blood coagulation abnormality, sympatho-adrenal response, and endothelial dysfunction [21, 22]. In the present study, the impact of HbA_1c_ variability on the risks of cardiovascular events in diabetic patients with preserved renal function remained significant after adjustments in the mean HbA_1c_. This suggests that HbA_1c_ variability may provide additional valuable information as a potential predictor of adverse cardiovascular outcomes.

Another important finding here is that the significant association between HbA_1c_ variability and cardiovascular events is not observed in patients with moderate-to-advanced CKD. This implies that the predictor power of HbA_1c_ variability on the risk of cardiovascular events among patients with CKD may be relatively lower. The prognostic role of HbA_1c_ variability in such patients is unclear because of impaired glucose metabolism in CKD, while the HbA_1c_ level may be altered by anemia or by the use of an erythropoiesis-stimulating agent. In patients with CKD, HbA_1c_ formation is reduced because the fragile red blood cell (RBC) has a shortened lifespan by 30–70% and carbamylated hemoglobin molecules in a uremic environment become resistant to glycosylation [23]. Young RBCs have a lower glycosylation rate compared to old RBCs, thereby altering HbA_1c_ formation [24]. Thus, although aggressive glycemic control may be beneficial for early cardiovascular disease prevention, there is paucity of data on outcomes supporting tight glycemic control in patients with CKD and HbA_1c_ variability may be a more useful indicator in preserved renal function for predicting clinical outcomes [25].

This study has several limitations. First, as an observational study, the number and frequency of HbA_1c_ measurement varied between individual patients. To minimize this, patients with fewer than three HbA_1c_ measurements during the follow-up period and those with a follow-up period shorter than 6 months were excluded. Second, we used DM duration > 5 years instead of continuous diabetes duration. The diabetes duration were always underestimated in the case of diabetes due to underdiagnosis. Referred to most of the studies, categorical division was used for DM duration and the diabetes complications were started as early as 5 years after the diagnosis of diabetes. So we preferred to follow most of the studies reported by using categorical division of ≦ and > 5 years in diabetes duration. In addition, post hoc analysis of the Action to Control Cardiovascular Risk in Diabetes (ACCORD) [26] and other trials suggest that cardiac autonomic neuropathy may be a predisposing factor to cardiovascular events. However, the patients here were not evaluated for heart rate variability and as such, the effect of cardiac autonomic neuropathy on cardiovascular events could not be assessed. Nonetheless, a diagnosis of diabetic neuropathy according to the ICD-9-CM code is included in the analysis. Lastly, the effect of medications on cardiovascular events was not evaluated because this study was not a clinical trial aimed at investigating the effects of medication. There is insufficient data on cumulative exposure duration and defined daily dose. In this study, the positive correlation between medications use and cardiovascular events may be due to selection bias.

In conclusion, greater HbA_1c_ variability is associated with increased risk of cardiovascular among patients with eGFR ≥ 60 min/ml/1.73m^2^, but not in patients with eGFR < 60 min/ml/1.73m^2^. These findings support the potential role of glucose variability, characterized by SD of HbA_1c_, as a predictor of cardiovascular events, independent of mean HbA_1c_ level, in patients with type 2 DM with preserved renal function. Clinicians should be cautious about fluctuations along with the direction of changes for preventing the development of adverse cardiovascular outcomes.

## Supporting information

S1 File Relevant data including HbA_1c_ variability with cardiovascular events in type 2 DM. (XLSX)
